# Tulp1 deficiency causes early-onset retinal degeneration through affecting ciliogenesis and activating ferroptosis in zebrafish

**DOI:** 10.1038/s41419-022-05372-w

**Published:** 2022-11-17

**Authors:** Danna Jia, Pan Gao, Yuexia Lv, Yuwen Huang, James Reilly, Kui Sun, Yunqiao Han, Hualei Hu, Xiang Chen, Zuxiao Zhang, Pei Li, Jiong Luo, Xinhua Shu, Zhaohui Tang, Fei Liu, Mugen Liu, Xiang Ren

**Affiliations:** 1 Key Laboratory of Molecular Biophysics of Ministry of Education, College of Life Science and Technology, Huazhong University of Science and Technology, Wuhan, Hubei 430074 PR China; 2 Prenatal Diagnosis Center, The Third Affiliated Hospital of Zhengzhou University, Zhengzhou, Henan 450052 China; 3 Department of Biological and Biomedical Sciences, Glasgow Caledonian University, Glasgow, UK; 4 State Key Laboratory of Freshwater Ecology and Biotechnology, Institute of Hydrobiology, The Innovative Academy of Seed Design, Hubei Hongshan Laboratory, Chinese Academy of Sciences, Wuhan, China; 5 University of Chinese Academy of Sciences, Beijing, 100049 China

**Keywords:** Retina, Disease model, Necroptosis, Ciliogenesis, Zebrafish

## Abstract

Mutations in TUB-like protein 1 (*TULP1*) are associated with severe early-onset retinal degeneration in humans. However, the pathogenesis remains largely unknown. There are two homologous genes of *TULP1* in zebrafish, namely *tulp1a* and *tulp1b*. Here, we generated the single knockout (*tulp1a*^*−/−*^ and *tulp1b*^*−/−*^) and double knockout (*tulp1-*dKO) models in zebrafish. Knockout of *tulp1a* resulted in the mislocalization of UV cone opsins and the degeneration of UV cones specifically, while knockout of *tulp1b* resulted in mislocalization of rod opsins and rod-cone degeneration. In the *tulp1-*dKO zebrafish, mislocalization of opsins was present in all types of photoreceptors, and severe degeneration was observed at a very early age, mimicking the clinical manifestations of *TULP1* patients. Photoreceptor cilium length was significantly reduced in the *tulp1-*dKO retinas. RNA-seq analysis showed that the expression of *tektin2* (*tekt2*), a ciliary and flagellar microtubule structural component, was downregulated in the *tulp1-*dKO zebrafish. Dual-luciferase reporter assay suggested that Tulp1a and Tulp1b transcriptionally activate the promoter of *tekt2*. In addition, ferroptosis might be activated in the *tulp1-*dKO zebrafish, as suggested by the up-regulation of genes related to the ferroptosis pathway, the shrinkage of mitochondria, reduction or disappearance of mitochondria cristae, and the iron and lipid droplet deposition in the retina of *tulp1-*dKO zebrafish. In conclusion, our study establishes an appropriate zebrafish model for *TULP1*-associated retinal degeneration and proposes that loss of TULP1 causes defects in cilia structure and opsin trafficking through the downregulation of *tekt2*, which further increases the death of photoreceptors via ferroptosis. These findings offer insight into the pathogenesis and clinical treatment of early-onset retinal degeneration.

## Introduction

*TULP1*, also known as Leber congenital amaurosis 15 (LCA15) or retinitis pigmentosa-14 (RP14) [1], has been associated with autosomal recessive early-onset retinal degeneration [2–8]. *TULP1* was first reported as a pathogenic gene of retinal degeneration in 1998 [2, 3]. At the present time, 99 pathogenic mutations have been reported (http://www.hgmd.cf.ac.uk/ac/gene.php?gene=TULP1). Most of the affected individuals display congenital nystagmus, night blindness, and severely reduced visual acuity in their first year of life [4, 6, 9]. Revealing the function of TULP1 is crucial for understanding the pathogenesis of *TULP1*-associated retinal degeneration.

TULP1 is a member of the tubby family proteins that contain a highly conserved tubby domain at the carboxyl-terminal. This protein family includes TUB, TULP1, TULP2, and TULP3 [10, 11]. Through a structure-directed approach, Boggon *et al*. predicted that Tubby-like proteins are a unique family of bipartite transcription factors [12]. Whether tubby proteins can regulate transcription under physiological conditions remains to be confirmed. In COS7 cells, TULP1 is localized to the plasma membrane and nucleus [13]. Another tubby family protein, tubby, is found primarily in the nucleus in a primary culture of hippocampal neurons [12]. In the retina, TULP1 is a photoreceptor-specific protein localized to the inner segments, connecting cilium, and synaptic terminals [14, 15]. Deletion of *Tulp1* in mice causes severe retinal degeneration and deficient protein transport in the photoreceptors [15–17]. The exact mechanism by which TULP1 regulates protein trafficking through the cilium is not well understood.

The photoreceptor cilium is supported by a microtubule-based axoneme backbone. Defects in the structure and/or function of photoreceptor cilium lead to a broad range of retinal dystrophy [18–20]. The involvement of TULP3 in ciliary protein transport has been extensively studied. TULP3 can directly bind GPCRs and facilitate their trafficking to the cilia [21]. TULP3 is also critical for cilia formation in hRPE-1 cells [22]. As for TULP1, a previous study demonstrated that the specific ciliary localization of photoreceptor disc component (PRCD), a protein involved in the formation of photoreceptor OS discs, is dependent on TULP1 and confirmed the interaction between PRCD and TULP1 [23]. Currently, the function of TULP1 and the exact etiology of how *TULP1* mutations cause early-onset retinal degeneration have not been clearly established.

Ferroptosis is characterized by the iron-dependent accumulation of lipid peroxidation [24]. In recent years, there has been increasing evidence that ferroptosis also plays a role in neurodegenerative diseases [25]. Treatment with iron chelators could increase the survival of photoreceptors in light-induced photoreceptor degeneration and retinitis pigmentosa mouse models [26, 27]. However, the possible involvement of ferroptosis in the death of photoreceptors in inherited retinal degeneration has not been fully studied.

In recent years, due to a number of benefits, zebrafish has become a popular animal model by which to study the pathogenic mechanism of retinal diseases [28]. In particular, in vitro fertilization and embryonic development of zebrafish allows real-time observation of organogenesis occurring at very early embryonic stages. Given the severe visual phenotype associated with *TULP1* patients from infancy, it is necessary to study the retinal changes before and after lesions. In this work, we generated a zebrafish *tulp1* knock-out model and found that Tulp1a and Tulp1b play an important role in the assembly of cilium in photoreceptors. Tulp1a and Tulp1b could increase the transcription activity of the *tekt2* promoter in ZF4 cells, suggesting that TULP1 functions as a transcription factor. In addition, our study highlights ferroptosis as a novel degeneration manner in early-onset retinal degeneration.

## Results

### Generation of the *tulp1a* and *tulp1b* knockout zebrafish line via CRISPR-Cas9 technique

Sequence alignment showed that there are two orthologous genes of *TULP1* in zebrafish, namely *tulp1a* and *tulp1b*. Zebrafish Tulp1a and Tulp1b show, respectively, about 72.45% and 70.94% alignment in the amino acid residues with human TULP1 (Fig. S1A). The predicted 3D structures of tubby domain show structural similarities between zebrafish and human (Fig. S1B). Our previous single-cell sequencing data on zebrafish retinas [29] showed that *tulp1a* and *tulp1b* are expressed in all types of photoreceptors, while *tulp1a* shows a higher expression than *tulp1b* in cones, particularly in the UV/blue cones (Fig. S2, Table S4).

**Fig. 1:**
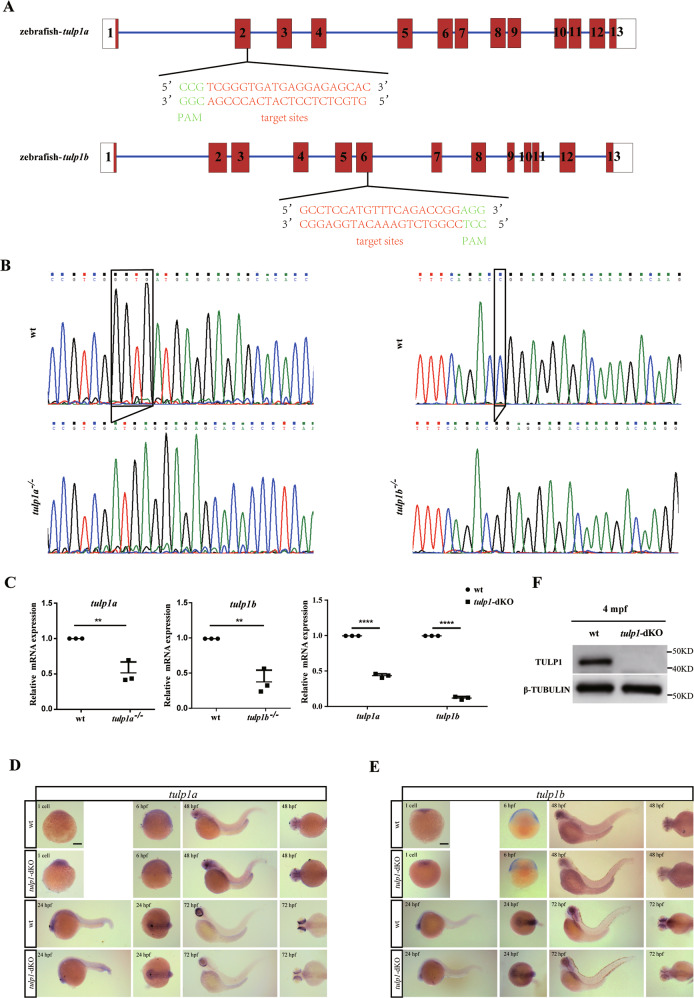
Generation of the *tulp1a*^*−/−*^ and *tulp1b*^*−/−*^ zebrafish lines. **A** The gene structure and CRISPR/Cas9 target sites are shown. The numbers represent exons. **B** DNA sequencing showing the *tulp1a* mutant line (*tulp1a*^*−/−*^) (c.115_118delGGTG), and *tulp1b* mutant line (*tulp1b*^−/−^) (c.591delC). **C** Decreased expression of *tulp1a* or *tulp1b* was detected by qRT-PCR in, respectively, *tulp1a*^−/−^, *tulp1b*^−/−^, and in *tulp1*-dKO zebrafish at 4 dpf. *18s-*rRNA was used as an endogenous control (*n* = 3). Mean ± SD. **P < 0.01. ****P < 0.0001. **D**, **E** Whole-mount in situ hybridization showing the expression pattern of *tulp1a* and *tulp1b* in wt and *tulp1*-dKO zebrafish. (*n* = 20). Scale bar: 50 µm. **F** The protein level of Tulp1 was undetectable at 4 mpf (*n* = 3). dpf: day post-fertilization; mpf: month post-fertilization.

The pathogenic mutations found in *TULP1* were distributed in all exons except for exon 9 (Fig. S1C). To explore the function of *TULP1*, we used CRISPR-Cas9 to generate knockout lines of *tulp1a* and *tulp1b*. The target sites aligned with the exon 4 and exon 8 of TULP1 in human, respectively (Fig. 1A, Fig. S1C). After three generations’ screening, we chose one *tulp1a* mutant line (*tulp1a*^*−/−*^) (c.115_118delGGTG, p.Gly39Metfs*12) and one *tulp1b* mutant line (*tulp1b*^−/−^) (c.591delC, p.Gly198Glufs*27) for subsequent study (Fig. 1B). A *tulp1a* and *tulp1b* double knockout zebrafish line (*tulp1*-dKO) was generated by crossing the two mutant lines. The mRNA levels of *tulp1a* and *tulp1b* were markedly reduced in, respectively, *tulp1a*^*−/−*^, *tulp1b*^*−/−*^, and in *tulp1*-dKO zebrafish (Fig. 1C). The temporal and spatial expression patterns of *tulp1a* and *tulp1b* were further determined by whole-mount in situ hybridization (WISH). At 24–48 hour post-fertilization (hpf), both genes were specifically expressed in the pineal gland. Subsequent to 48 hpf, *tulp1a* and *tulp1b* were observed to be expressed in the retina, whereas in *tulp1*-dKO zebrafish the expression of *tulp1a* and *tulp1b* was significantly decreased from 48 hpf onward (Fig. 1D, E). The Tulp1 protein could not be detected in *tulp1*-dKO zebrafish (Fig. 1F). Collectively, these results suggest our knockout zebrafish lines are successful.

### Photoreceptor degeneration in the *tulp1a* and *tulp1b* knockout zebrafish

Hematoxylin and Eosin staining results showed that no significant difference was observed in retina between wild-type (wt) and *tulp1a*^*−/−*^ zebrafish (Fig. S3A). To explore possible differences in photoreceptor types, we labeled rods and cones (red, green, blue, and UV cones) with specific antibodies (Rhodopsin, Opn1lw1, Opn1mw1, Opn1sw2, and Opn1sw1) in retinal sections. There are no differences in any of the other photoreceptor types except UV cones. In *tulp1a*^*−/−*^ zebrafish, at 3 day post-fertilization (dpf), the outer segments (OSs) of UV cones were shorter than those of wt zebrafish and were almost undetectable as early as 7 dpf (Fig. S3B and C). Western blot also showed no obvious difference in the expression of rod marker-Gnat1, even at 24 month post-fertilization (mpf), whereas the expression of cone marker-Gnat2 was slightly downregulated (Fig. S3D and E). These results indicate that only UV cones were degenerated in *tulp1a*^*−/−*^ zebrafish.

Compared to *tulp1a*^*−/−*^ mutants, significant photoreceptor degeneration was detected in *tulp1b*^*−/−*^ zebrafish, with the degree of degradation increasing with age. The outer nuclear layer (ONL) of *tulp1b*^*−/−*^ zebrafish was thinner and the OSs were shorter compared to those of wt zebrafish (Fig. S4A and B). Immunofluorescence results showed that in the *tulp1b*^*−/−*^ zebrafish, the length of rods OSs was obviously shorter as early as 3 dpf (Fig. S4C). Western blot showed that in *tulp1b*^−/−^ mutants the expression of Gnat1 was barely detectable at 1 mpf, while the downregulation of Gnat2 was not as significant as that of Gnat1 (Fig. S4D and E). These results suggest that *tulp1b* knockout led to a rod-cone degeneration phenotype.

**Fig. 2:**
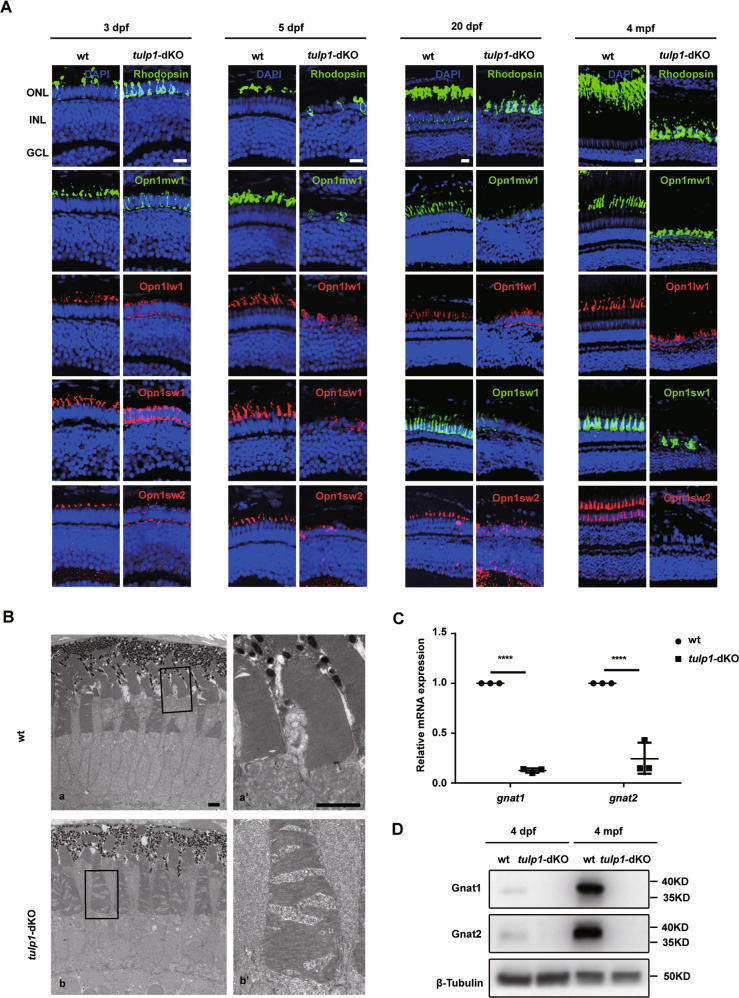
Photoreceptor degeneration and defective protein transport in *tulp1*-dKO zebrafish. **A** Sections were stained with rod-specific marker Rhodopsin, green cone-specific marker Opn1mw1, red cone-specific marker Opn1lw1, blue cone-specific marker Opn1sw2, and UV cone-specific marker Opn1sw1 at 3 dpf, 5 dpf, 20 dpf, and 4 mpf. ONL, outer nuclear layer; INL, inner nuclear layer; GCL, ganglion cell layer. Scale bar: 15 µm. **B** TEM of the photoreceptors of wt and *tulp1*-dKO zebrafish at 5 dpf. The higher-magnification images within the rectangles (in a and b) are shown on the right (a’ and b’). Scale bar: 2 µm. **C** Relative mRNA expression of *gnat1* and *gnat2* in wt and *tulp1*-dKO zebrafish at 4 dpf. Mean ± SD (*n* = 3). ****P < 0.0001. **D** Gnat1 and Gnat2 were detected by western blot.

Next, we examined the phenotype of the retina in *tulp1-*dKO zebrafish at the indicated ages. The signals of photoreceptor markers were markedly reduced at each age. By 5 dpf, the photoreceptor layer was almost absent from the central retina in *tulp1-*dKO zebrafish (Fig. 2A). The fine structure of photoreceptors was examined at 5 dpf using transmission electron microscopy (TEM). In wt retinas, membrane discs in OSs were well-organized. However, in *tulp1-*dKO retinas, the disc membranes were disorganized and contained concentric circular structures (Fig. 2B). Consistent with the above results, the mRNA and protein levels of Gnat1 and Gnat2 were significantly decreased (Fig. 2C and D). These results suggest that the function of photoreceptors was impaired at a very early stage in *tulp1-*dKO zebrafish, which is similar to the clinical phenotype of early-onset retinal degeneration [30].

### Ciliary defects precede photoreceptor degeneration in *tulp1-*dKO zebrafish

**Fig. 3:**
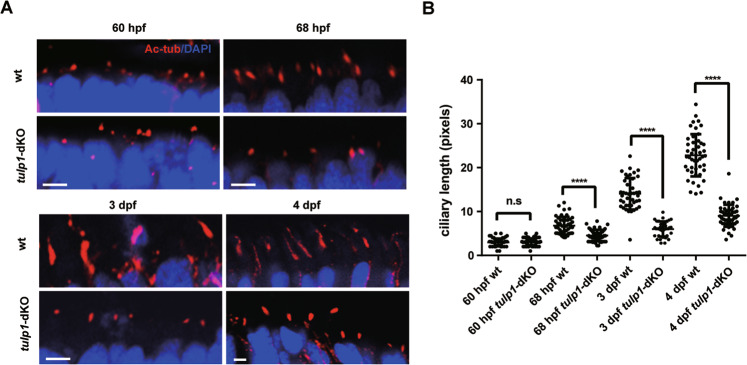
Defects in ciliogenesis of *tulp1*-dKO zebrafish. **A** Sections of wt and *tulp1*-dKO were stained with Ac-Tub at 60 hpf, 68 hpf, 3 dpf, and 4 dpf. Scale bar: 2 µm (*n* = 6). **B** Quantification of cilium lengths in wt and *tulp1*-dKO zebrafish presented in **A**. Mean ± SD. (*n* ≥ 40). ****P < 0.0001. Ac-Tub: Acetylated-α-Tubulin, the axonemal marker.

Given the mislocalization of Opn1sw1 in *tulp1a*^−/−^ zebrafish (Figure S3C), Rhodopsin in *tulp1b*^*−/−*^ zebrafish (Fig. S4B and C), and all opsins in *tulp1-*dKO zebrafish (Fig. 2A), it might be surmised that TULP1 has a function similar to that of TULP3 in mediating ciliary trafficking by interacting with diverse cargos [21]. Interestingly, no colocalization of Tulp1b and Rhodopsin was found in HEK293 cells (Fig. S5A). Moreover, Tulp1a and Tulp1b were localized in the nucleus in HEK293T cells, ARPE-19 cells, and ZF4 cells (Fig. S5B). These findings suggest that Tulp1a and Tulp1b may not affect the localization of Rhodopsin through direct interaction. Previous studies have demonstrated that TULP3 affected the formation of cilia [22]. To investigate the role of Tulp1 in cilia formation, ciliogenesis was analyzed in wt and *tulp1-*dKO zebrafish. The results showed that reduced ciliogenesis and shorter cilia were observed at 68 hpf, 3 dpf, and 4 dpf in *tulp1-*dKO zebrafish compared with wt (Fig. 3A, B), suggesting that ciliogenesis was affected in *tulp1-*dKO zebrafish.

### Tulp1a and Tulp1b affect ciliogenesis through promotion of *tekt2* expression

**Fig. 4:**
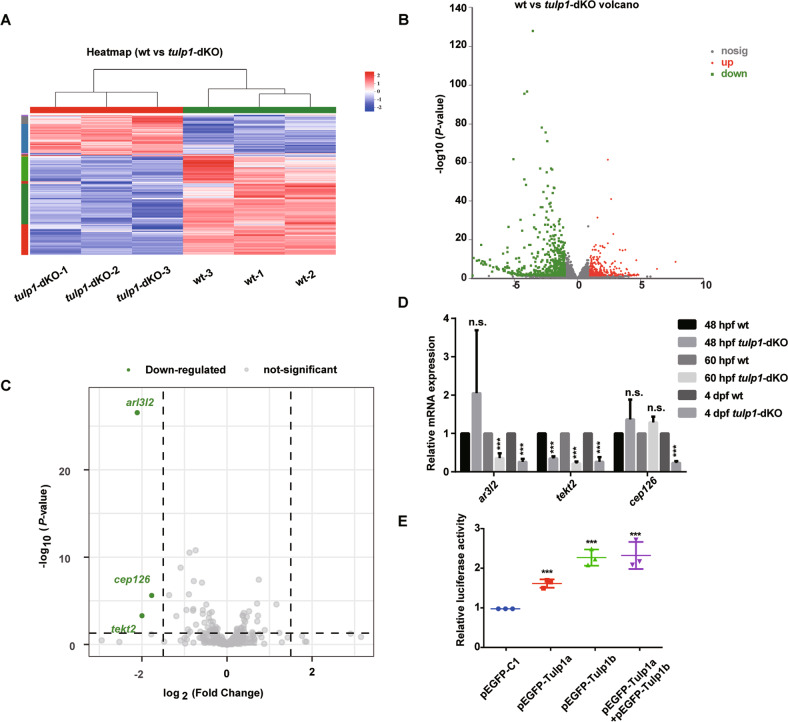
Tulp1a and Tulp1b regulate the expression of *tekt2*. Heatmap (**A**) and Volcano plot (**B**) for differential gene expression between wt and *tulp1*-dKO zebrafish. **C** Volcano plot displaying the gene expression related to cilium for wt versus *tulp1*-dKO. Genes with |log2FC| ≥ 1.5 and *P*-value ≤ 0.05 are highlighted in green. **D** The mRNA levels that significant enrichment in (**C**) were detected by qRT-PCR (*n* = 3). **E** Reporter plasmid containing the promotor of *tekt2* and pEGFP-C1, pEGFP-Tulp1a, pEGFP-Tulp1b were transfected into ZF4 cells (*n* = 3). Mean ± SD. ***P < 0.001.

To investigate the mechanism underlying the cilium defect and photoreceptor degeneration resulting from *tulp1* deletion, we performed RNA-seq of wt zebrafish and *tulp1-*dKO zebrafish. A total of 895 genes were expressed significantly differently between wt and *tulp1-*dKO zebrafish, of which 303 genes were significantly upregulated and 592 genes were significantly downregulated in the *tulp1-*dKO zebrafish (Fig. 4A, B). Interestingly, we found that only three genes related to cilium (*tekt2, arl3l2, cep126*) were significantly reduced in the RNA-seq data (Fig. 4C). Consistent with the RNA-seq data, a marked reduction in the expression of *tekt2, arl3l2,* and *cep126* was observed in the *tulp1-*dKO zebrafish at 4 dpf. Notably, however, only the expression of *tekt2* was significantly reduced as early as 48 hpf (Fig. 4D). Herein, we speculate that *tekt2* might be the target gene of Tulp1a and Tulp1b. The luciferase reporter assay results showed that Tulp1a and Tulp1b significantly increased the transcription activity of *tekt2* promoter in ZF4 cells. Co-expression of Tulp1a and Tulp1b further increased the activity of *tekt2* promotor in comparison with the expression of Tulp1a and Tulp1b separately (Fig. 4E). TEKT2 is a member of TEKTINs, a family of elongated proteins that assemble into extended filaments [31]. WISH showed that *tekt2* was indeed expressed in eyes (Fig. S6). The downregulation of *tekt2* may explain the shortened cilia length in *tulp1-*dKO zebrafish. Collectively, these results indicate that *tekt2* was downregulated in *tulp1-*dKO zebrafish and that Tulp1a and Tulp1b could promote the expression of *tekt2* in ZF4 cells.

### Ferroptosis was activated in photoreceptor cells of *tulp1*-dKO zebrafish

**Fig. 5:**
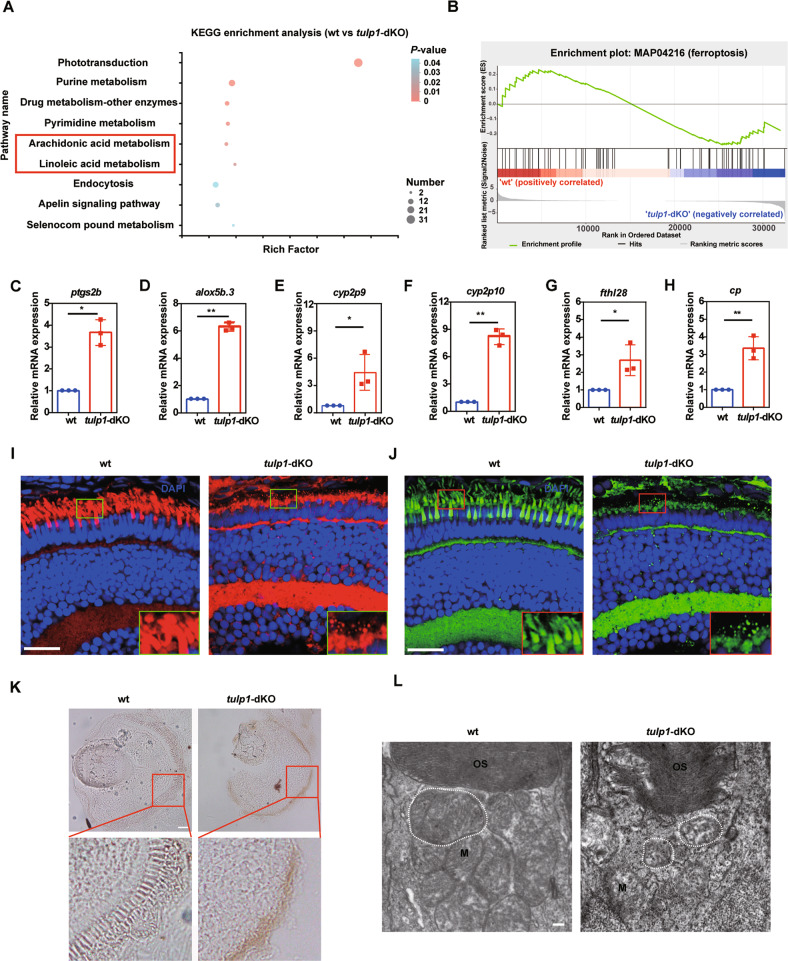
Ferroptosis signaling pathway was activated in *tulp1*-dKO zebrafish. **A** KEGG pathway enrichment analysis of the genes identified to be differentially expressed between wt and *tulp1*-dKO groups. The significantly enriched pathways are shown. **B** GSEA of ferroptosis-related genes. **C**–**H** Relative expression of genes involved in ferroptosis was detected by qRT-PCR at 4 dpf. Mean ± SD. *P < 0.05. **P < 0.01. Lipid droplets performed by Nile Red (**I**) or BODIPY (**J**) in wt and *tulp1*-dKO zebrafish at 4 dpf. Enlarged images of the boxed areas are shown in the lower right corner. Scale bar: 20 µm. **K** Perls/DAB staining in wt and *tulp1*-dKO zebrafish at 4 dpf. Enlarged images of the boxed areas are shown beneath. Scale bar: 25 µm. **L** TEM images of mitochondria from wt and *tulp1*-dKO zebrafish. M, mitochondria. OS, outer segment. Scale bar: 0.2 µm.

As shown in Fig. 2A, there were severe degeneration of photoreceptors in *tulp1-*dKO zebrafish. To investigate the mechanism of cell death, we performed Kyoto Encyclopedia of Genes and Genomes (KEGG) pathway enrichment analysis based on the RNA-seq data. KEGG analysis revealed that the phototransduction pathway is significantly enriched, which is consistent with the retina degeneration phenotype in *tulp1-*dKO zebrafish. Interestingly, the Arachidonic acid metabolism and Linoleic acid metabolism pathway were significantly enriched (Fig. 5A), which were considered to be involved in the ferroptosis [24, 32]. The Gene Set Enrichment Analysis (GSEA) also showed a tight correlation between ferroptosis and the *tulp1-*dKO gene sets (Fig. 5B). The iron-containing enzyme lipoxygenase is the main promotor of ferroptosis through the production of lipid hydroperoxides [33]. The expression of genes involved in the iron homeostasis and lipid peroxidation was verified by qRT-PCR (Fig. 5C–H). Consistent with RNA-seq data, the expression of *ptgs2b*, *alox5b.3*, *cyp2p9*, *cyp2p10*—the in vivo biomarkers for ferroptosis that contributes to lipid peroxidation [33, 34]—was significantly increased in the *tulp1-*dKO zebrafish (Fig. 5C–F). Furthermore, accumulation of lipid, detected using BODIPY and Nile red staining, was observed in the *tulp1-*dKO retina (Fig. 5I, J). In addition, we also detected iron accumulation by Perls/DAB staining. The results showed that there was markedly increased iron signaling in the photoreceptors of *tulp1-*dKO zebrafish compared with wt zebrafish at 4 dpf (Fig. 5K). Consistent with this iron accumulation, the expression of *fthl28* and *cp* was upregulated in the *tulp1-*dKO zebrafish (Fig. 5G, H), suggesting a disruption in iron homeostasis [35–37]. ATF3 contributed to ferroptosis via increasing H_2_O_2_ and iron [38], while the expression of *atf3* was upregulated in the *tulp1-*dKO zebrafish, suggested the activation of ferroptosis in *tulp1-*dKO zebrafish (Fig. S7). Morphology of mitochondria is also one of the features of ferroptosis [32]. TEM results showed mitochondria exhibited changes characteristic of ferroptosis, including shrinkage and disappearance of cristae in the inner segments of *tulp1-*dKO zebrafish compared with wt zebrafish (Fig. 5L). However, the expression levels of Gpx4a and Gpx4b were unchanged as detected by qRT-PCR and western blot (Fig. S8). Based on the above, we conclude that ferroptosis participates in the photoreceptor degeneration seen in *tulp1-*dKO zebrafish.

## Discussion

**Fig. 6:**
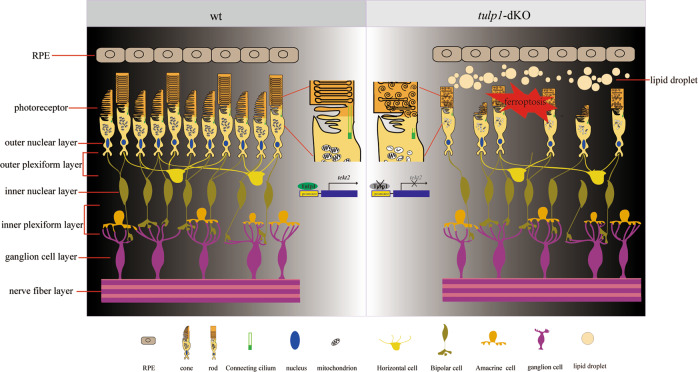
Schematic summary of the main findings in this study. Depletion of Tulp1a and Tulp1b in zebrafish diminished the transcriptional activity of *tekt2*, which impaired the ciliogenesis of photoreceptors, and thus interfering the trafficking of opsins. Eventually, ferroptosis was activated in photoreceptors, and retina was degenerated.

Shortened cilium in photoreceptors impedes the transporting of opsins and leads to the mislocalization in *tulp1-*dKO zebrafish. RNA-seq analysis and subsequent experiments suggest that downregulation of *tekt2* might be the cause of the shortened cilium and indicates an important role of ferroptosis in photoreceptor degeneration. This is the first time that the effect on cilia formation of TULP1 depletion has been elucidated. In addition, in vitro luciferase assay confirmed the possibility of TULP1 as a transcription factor (Fig. 6).

Loss of TULP1 results in severe early-onset retinal degeneration. Although many recent studies have explored the function of TULP1, there are some problems that remain unresolved: (1) Tubby proteins were predicted to be the transcription factors [12, 39], but the direct evidence for their role as transcription factors has not been reported; (2) Protein mislocalization has been reported in *Tulp1* knockout mice, although how *TULP1* mutation causes protein mislocalization has not been studied; (3) The mechanism of photoreceptor degeneration in *TULP1* mutant patients is not clear.

In the present study, we utilized the zebrafish to investigate the function of TULP1 and to explore the above questions. In *tulp1a*^*−/−*^ zebrafish, we observed, specific mislocalization of opsins in UV cones that then suffered degeneration. In *tulp1b*^*−/−*^ zebrafish, there was a retinitis pigmentosa phenotype. The different phenotypes in the two models may be due to the different expression levels of the two genes in different photoreceptor cells, a possibility that can be resolved through further study. Lital et al. showed that TULP1 is required for specific localization of PRCD by direct interaction [23]. In contrast, in the present study, no colocalization of Tulp1b and Rhodopsin was found, suggesting that Tulp1 does not mediate Rhodopsin transport by direct interaction. Cross-sectional views of cilium had the normal 9 + 0 arrangement of microtubule pairs in mice [15], suggesting that the basal body of cilium is normal. In our study, we demonstrated that *tulp1a* and *tulp1b* affected the length of cilium in photoreceptors and caused subsequent mislocalization of proteins. Recently, Hong *et al*. suggested that TULP1 transfection could not rescue cilia formation and length in *TULP3* KO RPE1 cells [22], a finding that is not consistent with our results probably due to differential requirements of TULP1 for ciliogenesis in each cell type.

Tekt2 is a member of Tektins, originally identified as structural components of axonemal doublet microtubules in cilia and flagella [31]. Human TEKT2 has been suggested to be an indicator of sperm motility loss [40]. Rebecca et al. demonstrated that one member of this family, Tektin-1, is a positive regulator of cilium length [41]. Our analysis showed that *tekt2* was downregulated prior to the emergence of shortened cilium, fueling our speculation that *tekt2* has specific functions in ciliogenesis in the photoreceptor. As Tulp1a and Tulp1b indeed enhanced the transcriptional activity of the *tekt2* promoter, we surmise that *tekt2* is one of the downstream effectors of Tulp1a and Tulp1b in zebrafish. However, the direct interaction and other target genes require further investigation. These results also indicated that *tekt2* might be a candidate gene of retinal disease.

Iron dyshomeostasis and lipid peroxidation are features of ferroptosis. In addition to being a cofactor of iron-containing lipoxygenase, iron can also generate free radicals directly through the Fenton reaction, leading to lipid peroxidation [42, 43]. In our study, iron accumulation, lipid droplets, and abnormal mitochondria in retina were detected in the *tulp1-*dKO zebrafish. The genes involved in lipid peroxide, such as *ptgs2b*, *alox5b.3, cyp2p9, cyp2p10*, were considerably upregulated. Together, these results indicate that ferroptosis contributes to the death of photoreceptors in *tulp1-*dKO zebrafish. However, what causes the iron accumulation is unclear. Based on our own and other studies, we speculate that the disturbed visual cycle and phototransduction cascade may cause the disorder of iron homeostasis, as the enzymes involved in these two processes such as RPE65, GC, GCAP5 are iron-dependent [44]. In addition, abnormal connection between RPE and photoreceptors cause disordered iron homeostasis, as that iron is recycled through phagocytosis [45]. As for lipid accumulation: with the rapid degradation of photoreceptors, a large number of outer segments (containing high levels of polyunsaturated fatty acids), which could not be phagocytized and digested by RPE cells in a timely fashion, accumulated in the photoreceptor layer, so resulting in lipid accumulation. Caberoy *et al*. suggested that Tulp1 could facilitate phagocytosis through MerTK [46], such that knockout Tulp1 may decrease the effect of RPE phagocytosis. Masek et al. revealed that loss of the Bardet-Biedl syndrome 1 (important for ciliary transport) in zebrafish locally disrupted lipid homeostasis [47]. Here, in our model, abnormal ciliary transport may lead to disturbed lipid homeostasis. GPX4 is a central inhibitor of ferroptosis, GPX4 degradation or inhibition can promote ferroptosis [48]. The reason why the expression of Gpx4 was unaffected in our zebrafish *tulp1*-dKO model remains unclear. Possibly, there may another GPX family protein or GPX4-independent systems responsible for the inhibition of ferroptosis in retinal cells during retinal degeneration. Further studies are needed to demonstrate this assumption in the future. Given that iron-mediated ferroptosis is involved in the death of photoreceptors in the *tulp1-*dKO zebrafish, it is plausible that an iron-chelating agent may be useful in treatment. Our current study also raises an intriguing question as to a possible role of ferroptosis in retinal diseases caused by other genes.

In conclusion, we revealed an essential role of TULP1 in ciliogenesis and survival of photoreceptors. Our results showed that Tulp1a and Tulp1b might act as transcription factors to regulate the expression of *tekt2* to affect ciliogenesis. The *tulp1-*dKO zebrafish line may be extremely useful in providing further insights into the physiological function of TULP1 in ciliogenesis and the role of ferroptosis in inherited retinal degeneration, and therefore act as a basis for new strategies for the prevention and treatment of such diseases.

## Materials and methods

### Zebrafish maintenance and breeding

The zebrafish were kept in the recirculating water system (pH 6.6–7.4, 26–28.5 °C) with a daily cycle of 14 h of light and 10 h of dark. The zebrafish embryos were collected and placed in E3 medium and kept in a 28.5 °C incubator until hatched. To avoid bias affecting the selection of zebrafish, we randomized the embryos for all experiments, no blinding was used. There were no inclusion or exclusion criteria used in the selection of the animals. All studies were approved by the Ethics Committee of Huazhong University of Science and Technology.

### Generation of zebrafish knockout lines

The CRISPR/Cas9 system was purchased from the China Zebrafish Resource Center (CZRC). CRISPR/Cas9 target sites were designed using the online tool CHOPCHOP (http://chopchop.cbu.uib.no/) [49]. The single-guide RNAs (sgRNAs) and Cas9 mRNA were synthesized using the in vitro transcription kits (Thermo Scientific and Ambion). Wild-type (wt) zebrafish embryos at the one-cell stage were co-injected with the Cas9 mRNA and sgRNAs. The detailed experimental methods have been described previously [50].

### Hematoxylin and eosin (H&E) staining

The eyes of zebrafish were fixed with 4% PFA for 3 h at room temperature (RT), dehydrated with 30% sucrose overnight at 4 °C, and then embedded in OCT and sectioned as described in previous studies [51]. The sections were stained with the Hematoxylin and Eosin Staining Kit (Beyotime, Shanghai, China). Images were captured by optical microscope BX53.

### Immunofluorescence

The frozen sections were dried at RT, soaked in PDT (PBS containing 1% DMSO and 0.1% TritonX-100) twice for ten minutes to remove the OCT, then blocked with PBDT (PDT containing 1%BSA) containing 10% goat serum for 1 h at RT. After removing the blocking solution, the slides were incubated with the primary antibody overnight at 4 °C (Nile Red and BODIPY staining were incubated for 10 min at RT). The slides were washed three times with PDT and incubated with the secondary antibody of the corresponding species for 1 h at 37 °C in the dark. Following by staining the cell nucleus with DAPI and washing with PBS, the sections were mounted with 50% glycerol in PBS. The fluorescence images were captured with a confocal laser-scanning microscope (FV3000, Olympus). The list of the primary antibodies is provided in Table S1.

### Western blot

About 35 heads of zebrafish larvae age of <7 day post-fertilization (dpf) or three eyes of adult fish were collected and sonicated in RIPA Lysis Solution (Beyotime, Shanghai, China). The loading buffer was added and the protein samples were boiled for 10 min and then cooled on ice for 5 min. Protein concentration was measured with the BCA Protein Assay Kit (Beyotime, Shanghai, China). Western blot was performed as described previously [52]. The membranes were rinsed with SuperSignal® ELISA FemtoMaximum Sensitivity Substrate (Thermo Scientific, USA) and pictures were captured with ChemiDoc XRS + imaging system (Bio-Rad Laboratories). The list of antibodies used in the western blot is provided in Table S1.

### Real-time fluorescence quantitative PCR (qRT-PCR)

Heads of the embryos were used for total RNA extraction. For each sample, 35 embryos were used. Total RNA was extracted with the RNA isolater Total RNA Extraction Reagent (Vazyme Biotech, Nanjing, China). The cDNA was synthesized using the HiScript II Q RT SuperMix for qPCR (+gDNA wiper) kit (Vazyme Biotech, Nanjing, China) according to the manufacturer’s manual. qRT-PCR was performed using the AceQ™ qPCR SYBR Green Master Mix (Vazyme Biotech, Nanjing, China) on the StepOnePlus™ real-time PCR system (Life Technologies). Data were analyzed with the GraphPad 5.1 software. Primers used for qRT-PCR are listed in Table S2.

### Transmission electron microscopy (TEM)

For ultrastructural analysis, zebrafish embryos at 5 dpf were collected and processed as previously described [53]. The embryos were fixed in 2.5% glutaraldehyde (Servicebio, Wuhan, China) overnight at 4 °C. After three washes with PBS buffer, the embryos were further fixed in 1% osmium tetroxide for 2 h at RT. Next, the embryos were gradient dehydrated with ethanol and incubated in acetone for 20 min at RT. After treatment with propylene oxide, the embryos were embedded in epoxy resin. Then Reichert-Jung ultramicrotome was then used to produce ultrathin slices. Images were taken using the transmission electron microscope (HT7700, Hitachi, Japan).

### Whole-mount in situ hybridization

Whole-mount in situ hybridization (WISH) was performed as previously described [54]. In brief, all DNA templates for the RNA probes were amplified from the cDNA library of whole embryos at 4 dpf, and cloned into the pGEM®-T Easy plasmid (Promega, A1360). The sequences of the in vitro transcription templates were validated by Sanger sequencing. The RNA probes were transcribed using the MAXIscript™ SP6/T7 Transcription Kit (Invitrogen, USA). The primer sequences used to amplify the DNA templates for synthesizing RNA probes were as follows: *tulp1a*: forward, AAAGAAGAAAGGCAAAGG; reverse, GTATCGGCACTCGCTCA. *tulp1b*: forward, AAAGAAAGCGGCCAAATCCGA; reverse, CGGGTCACTTTGCACTTCAC. *tekt2*: forward, GCCGGGCAAGGGAAGAGATG; reverse, AGCGCTGCCGGGTCTGGT.

### Perls/DAB staining

The retinal frozen sections were treated with 30% hydrogen peroxide solution to remove pigment and soaked in PBS 3 times for 2 min each. The sections were then incubated with Perls reagent (5% potassium ferrocyanide (Sigma, P3289) and 5% HCl in ddH_2_O) for 1 h at 25 °C. After removing the Perls reagent, the sections were washed with ddH_2_O 3 times for 2 min each. Sections were treated with DAB solution (Servicebio, China) for 30 min to amplify the signals, and then washed with ddH_2_O 3 times for 5 min each. Finally, sections were mounted with 90% glycerol in PBS (PH 7.6) and imaged with optical microscope BX53.

### Cell culture, plasmid construction, and Luciferase Reporter Assay

ZF4 cells were raised using DMEM-12 (Dulbecco’s Modified Eagle Medium/Nutrient Mixture F-12, Gibco, Grand Island, New York, USA) containing 10% fetal bovine serum (FBS, Biological Industries) at 28.5 °C in the presence of 5% CO_2_. The full-length coding sequence of *tulp1a* and *tulp1b* were separately subcloned into the pEGFP-C1 vector (Miaolingbio, P0134). The promotor region (2.1 kb) of *tekt2* was subcloned into the pGL3-basic luciferase reporter vector. Primers are listed in Table S3. Plasmids were transfected into ZF4 cells with X-tremeGENE HP DNA Transfection Reagent (Roche) in OMEM (Opti-MEMI Reduced Serum Medium, Gibco). The medium was changed to DMEM-12 containing 10% FBS after 8 h incubation. After 36 h, the cells were harvested and the activity of luciferase was examined using the Dual Luciferase® Reporter Assay System (Promega, Madison, Wisconsin, USA). The experiments of HEK293, HEK293T, and ARPE-19 cells were performed as described previously [53, 55]. All cells were routinely tested for mycoplasma at regular intervals throughout the whole course of the study.

### RNA-seq and bioinformatics analysis

The heads of zebrafish larvae at 4 dpf were dissected for RNA extraction. Nanodrop2000 was used to measure the concentration and purity of the RNA samples. Agarose gel electrophoresis and Agilent were used to estimate the integrity of RNA. RNA-Seq was performed on Illumina Novaseq 6000 platform by Majorbio (Shanghai, China). Sequencing data were mapped to the reference genomes (the zebrafish GRCz11 genome) using HISAT2 software [56, 57]. RSEM was used to obtain the Read Counts of each gene/transcript by comparing the results to the genome and the genome annotation file, and then TPM conversion was performed to obtain the standardized gene/transcript expression level [58]. Differentially expressed (DE) genes were determined by the R package DESeq2 using the following cut-off values: FC ≥ 2 and adjusted *P*-value ≤ 0.05. KEGG (Kyoto Encyclopedia of Genes and Genomes) pathway enrichment analysis was performed on DE genes using scripts written in R language with default parameters. The KEGG pathway is considered as significantly enriched if *P*-value ≤ 0.05. Gene Set Enrichment Analysis (GSEA) and Volcano analysis were performed using the online Hiplot (https://hiplot.com.cn/) [59].

### Statistical analysis

All data were analyzed with a two-tailed *t*-test by using the GraphPad Prism 6 software and represented as mean ± SD. All the experiments were independently repeated at least three times. *P*-value was used to indicate significance: *P < 0.05, **P < 0.01, ***P < 0.001 and ****P < 0.0001. n.s (no significance) *P* > 0.05.

## Supplementary information

Supplemental Material

Original western blots

aj-checklist

Table S1

Table S2

Table S3

Table S4

## Data Availability

The NGS data are available under the GEO accession numbers: GSE214642
