# Supplementary material for: Tulp1 deficiency causes early-onset retinal degeneration through affecting ciliogenesis and activating ferroptosis in zebrafish
Source: Cell Death Dis. 2022 Nov 17;13(11):962. doi: 10.1038/s41419-022-05372-w (PMC9672332; doi:10.1038/s41419-022-05372-w)
Supplement: Supplementary file 1 — Supplemental Material [file 41419_2022_5372_MOESM1_ESM.docx]

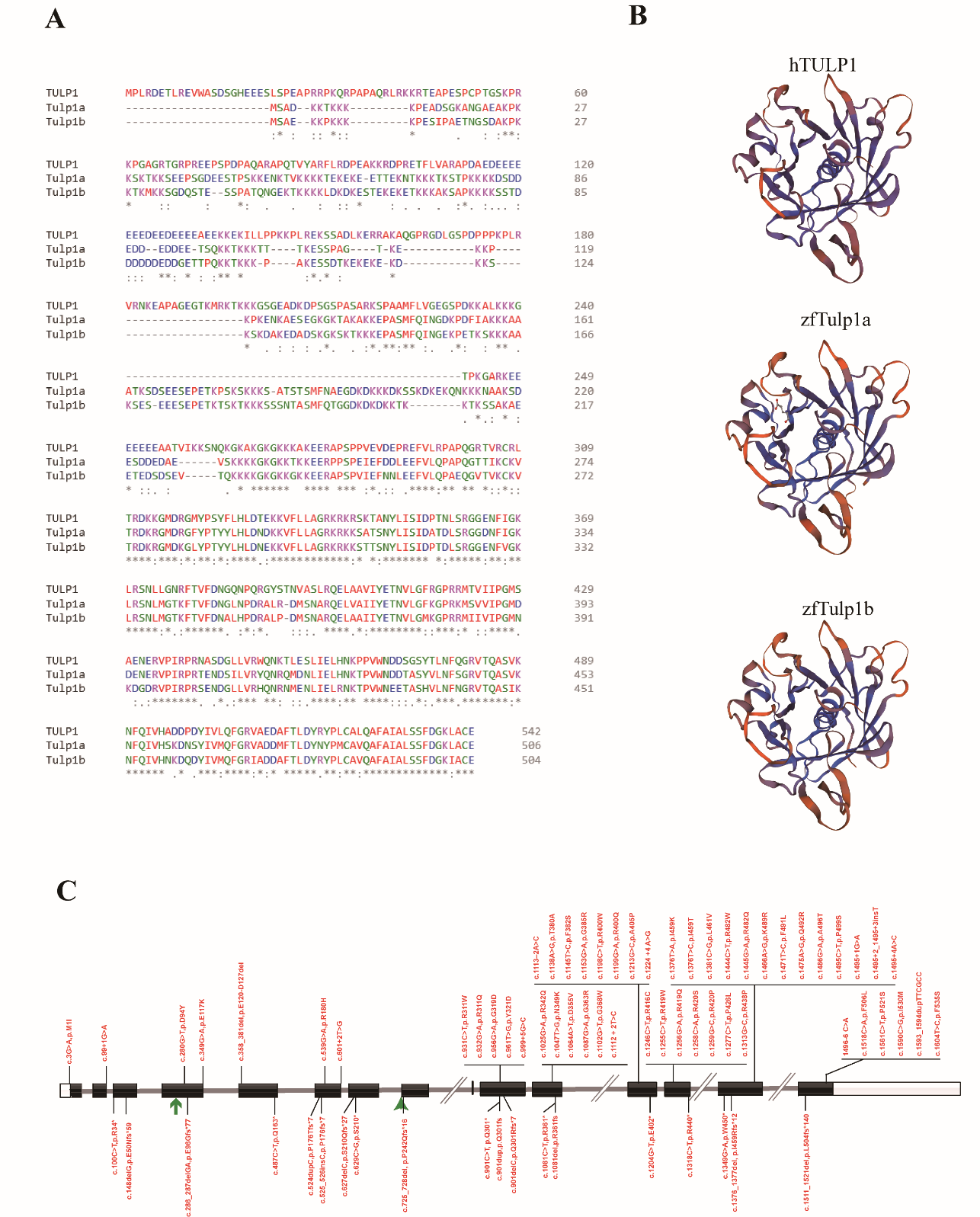


**Figure S1** Protein conservation analysis of TULP1 in human and zebrafish. (A) Sequence alignment showing the conserved regions in TULP1 between human and zebrafish. (B) Three-dimensional molecular structure of tubby domain of TULP1 in human or zebrafish. (C) The schematic representation of pathogenic mutations found in *TULP1* ([NM_003322.3](http://www.ncbi.nlm.nih.gov/entrez/viewer.fcgi?val=NM_003322.3)). The green arrow and arrowhead indicate the mutant target align with the *tulp1a* and *tulp1b*, respectively.


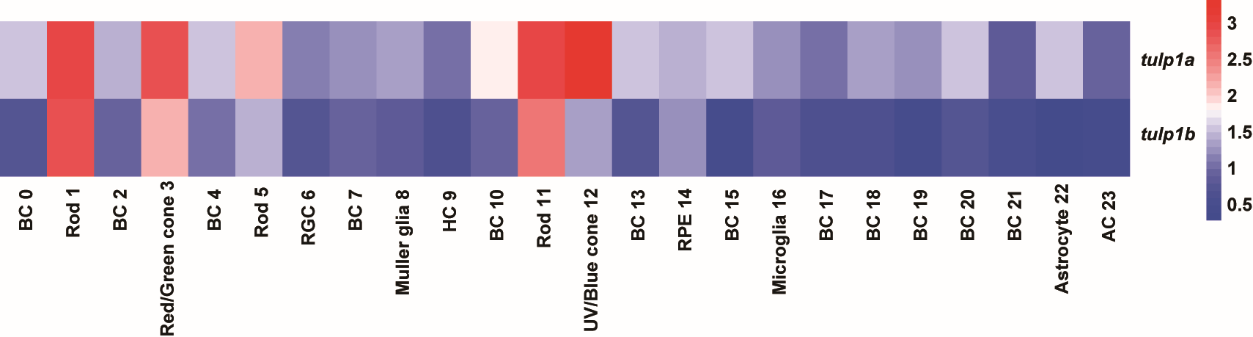


**Figure S2** Clustering analysis of the expression pattern of *tulp1a* and *tulp1b* in the retina by using data from single-cell sequencing data. AC, amacrine cells; BC, bipolar cells; RPE, retinal pigment epithelium; HC, horizontal cells; RGC, retinal ganglion cells.


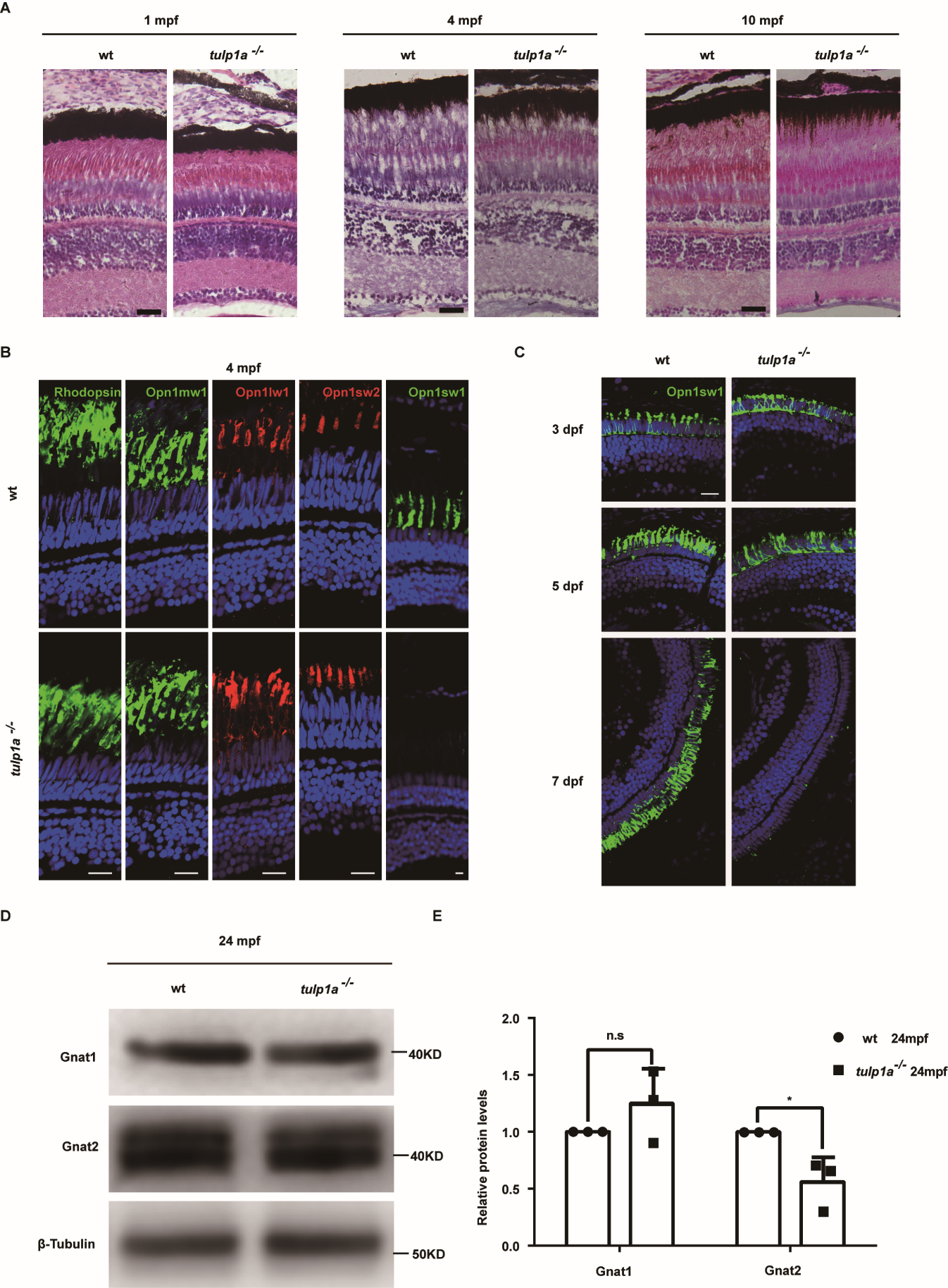


**Figure S3** The UV cone degeneration in *tulp1a^-/-^* zebrafish. (A) HE staining of wt and *tulp1a^-/-^* zebrafish at 1mpf, 4mpf, and 10 mpf. Scale bar: 25 µm. (B) The photoreceptors of wt and *tulp1a^-/-^* zebrafish were labeled with specific markers at 4mpf. Nuclei were labeled with DAPI (blue). Scale bar: 15 µm. (C) The UV cones of wt and *tulp1a^-/-^* zebrafish were labeled with Opn1sw1 at 3, 5 and 7 dpf. wt: wild-type. Scale bar: 15 µm. (D) Protein levels of Gnat1 and Gnat2 were detected in wt and *tulp1a*^-/-^ zebrafish at 24 mpf. (E) Relative protein levels of Gnat1 and Gnat2 presented in D. Mean ± SD. * *P* < 0.05, n.s (No Significance) *P* > 0.05.


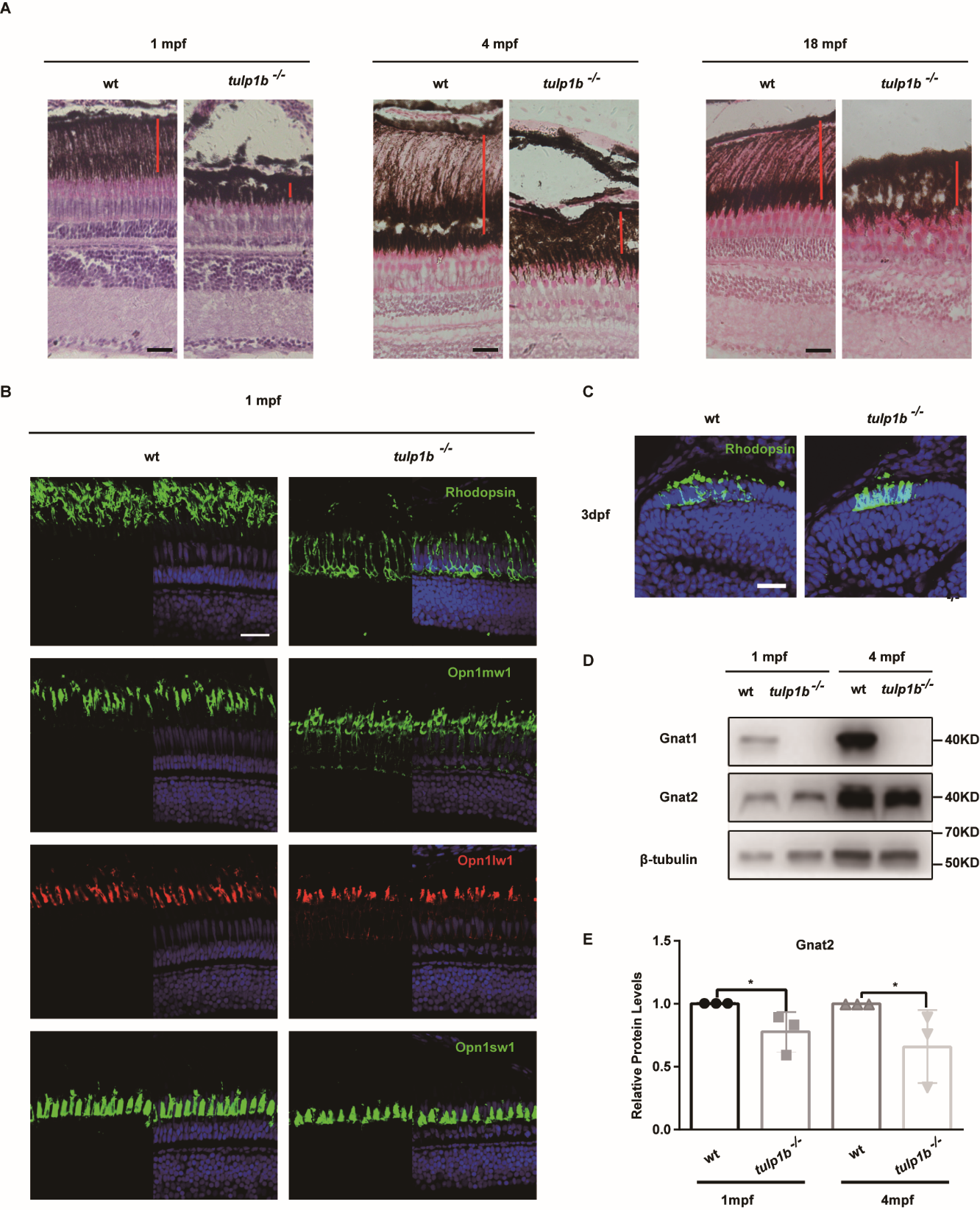


**Figure S4** Progressive retina degeneration was observed in *tulp1b* knockout zebrafish (A) HE staining of wt and *tulp1b^-/-^* zebrafish at 1mpf, 4mpf, and 18 mpf. The red lines indicate the outer segments of cones. Scale bar: 25 µm. (B) Cryosections of wt and *tulp1b^-/-^* were stained with Rhodopsin, Opn1mw1, Opn1lw1, Opn1sw1 at 1mpf. Scale bar: 25 µm. (C) The sections were stained with Rhodopsin in wt and *tulp1b^-/-^* zebrafish at 3dpf. Scale bar: 20 µm. (D) Protein levels of Gnat1 and Gnat2 were detected by western blot. (E) Relative levels of Gnat2 presented in D. Mean ± SD. (n = 3). * *P* < 0.05.


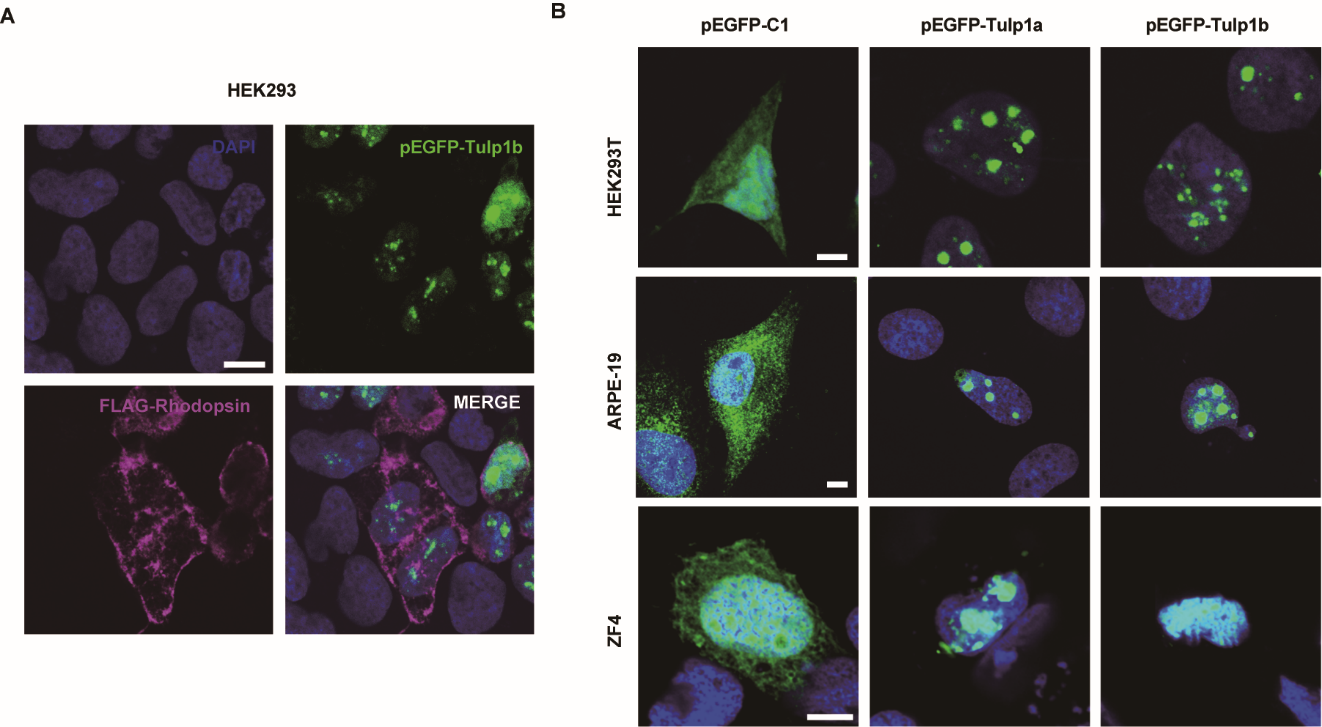


**Figure S5** Tulp1a and Tulp1b are localized in the nucleus. (A) Subcellular localization of Tulp1b and Rhodopsin in HEK293 cells. Scale bar: 2 µm. (B) Subcellular localization of Tulp1a and Tulp1b in HEK293T cells, ARPE-19 cells, and ZF4 cells. Nuclei were labeled with DAPI (blue). Scale bar: 5 µm.


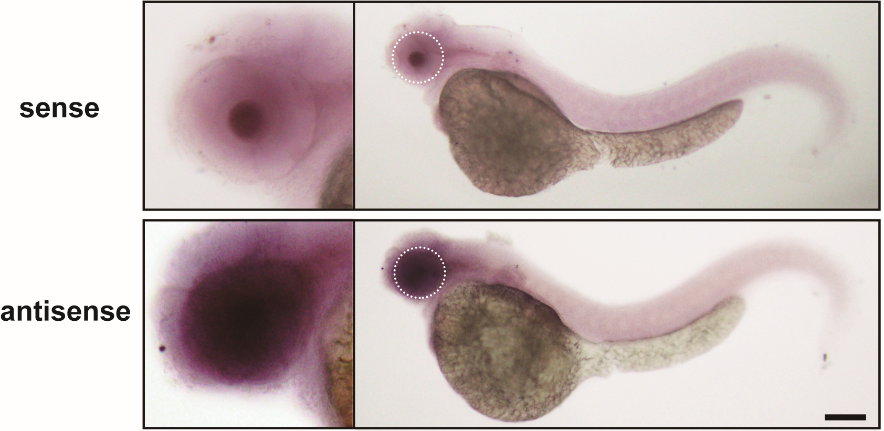


**Figure S6** The expression of *tekt2* was detected by WISH at 48h. The dashed circles show the eyes (n = 20). Scale bar: 100 µm.

­
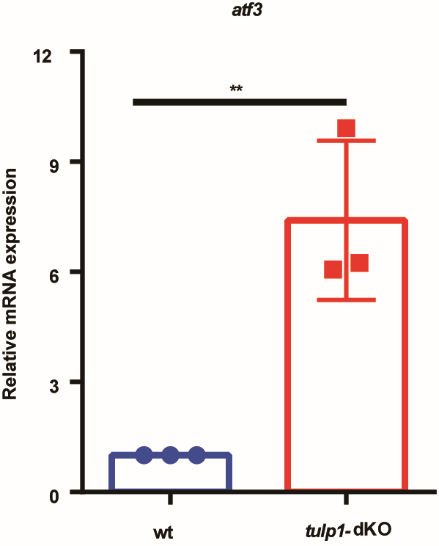


**Figure S7** The expression level of *atf3* was detected by qRT-PCR. Mean ± SD. (n = 3). ** *P* < 0.01.

**
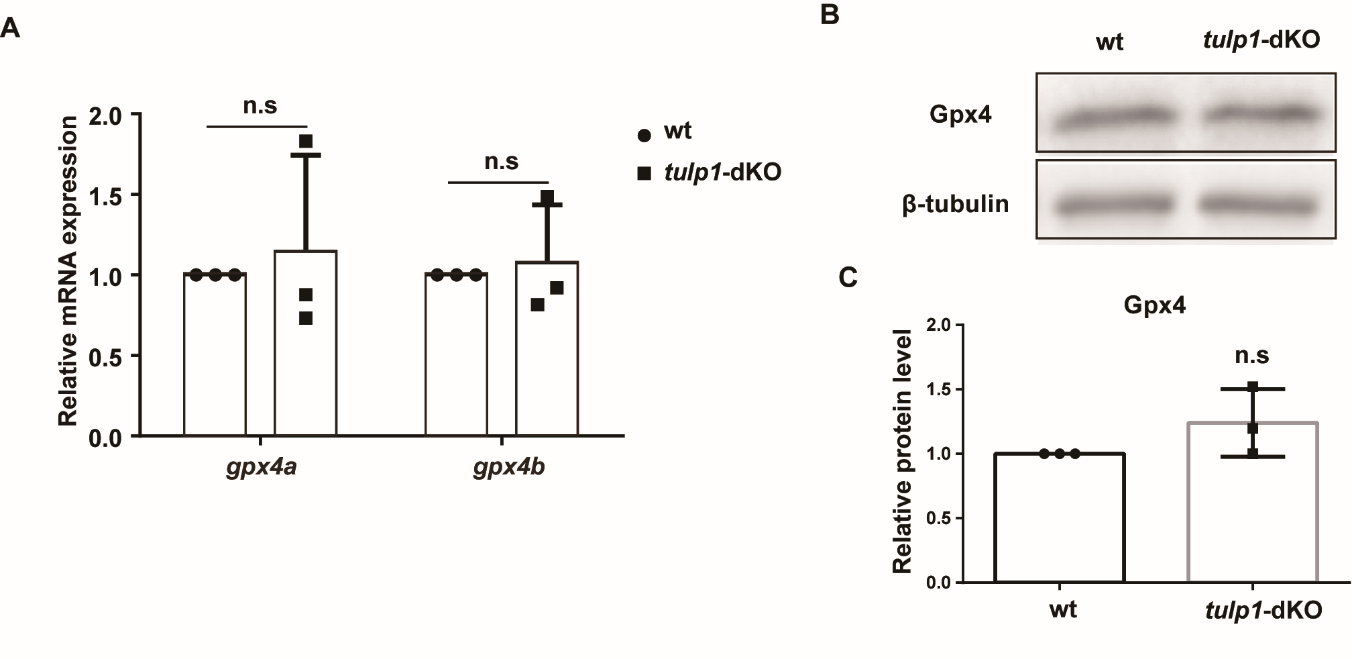
**

**Figure S8** The expression of Gpx4 was not affected in *tulp1*-dKO zebrafish. (A) The expression levels of Gpx4a and Gpx4b was detected by qRT-PCR. *18s* was used as an internal control. (B) Protein level of Gpx4 was detected by western blot. β-tubulin was used as an internal control. (C) Relative level of Gpx4 presented in B. Mean ± SD. (n = 3). n.s (No Significance), P > 0.05.
