# Supplementary figures and images for: Tulp1 deficiency causes early-onset retinal degeneration through affecting ciliogenesis and activating ferroptosis in zebrafish
Source: Cell Death Dis. 2022 Nov 17;13(11):962. doi: 10.1038/s41419-022-05372-w (PMC9672332; doi:10.1038/s41419-022-05372-w)

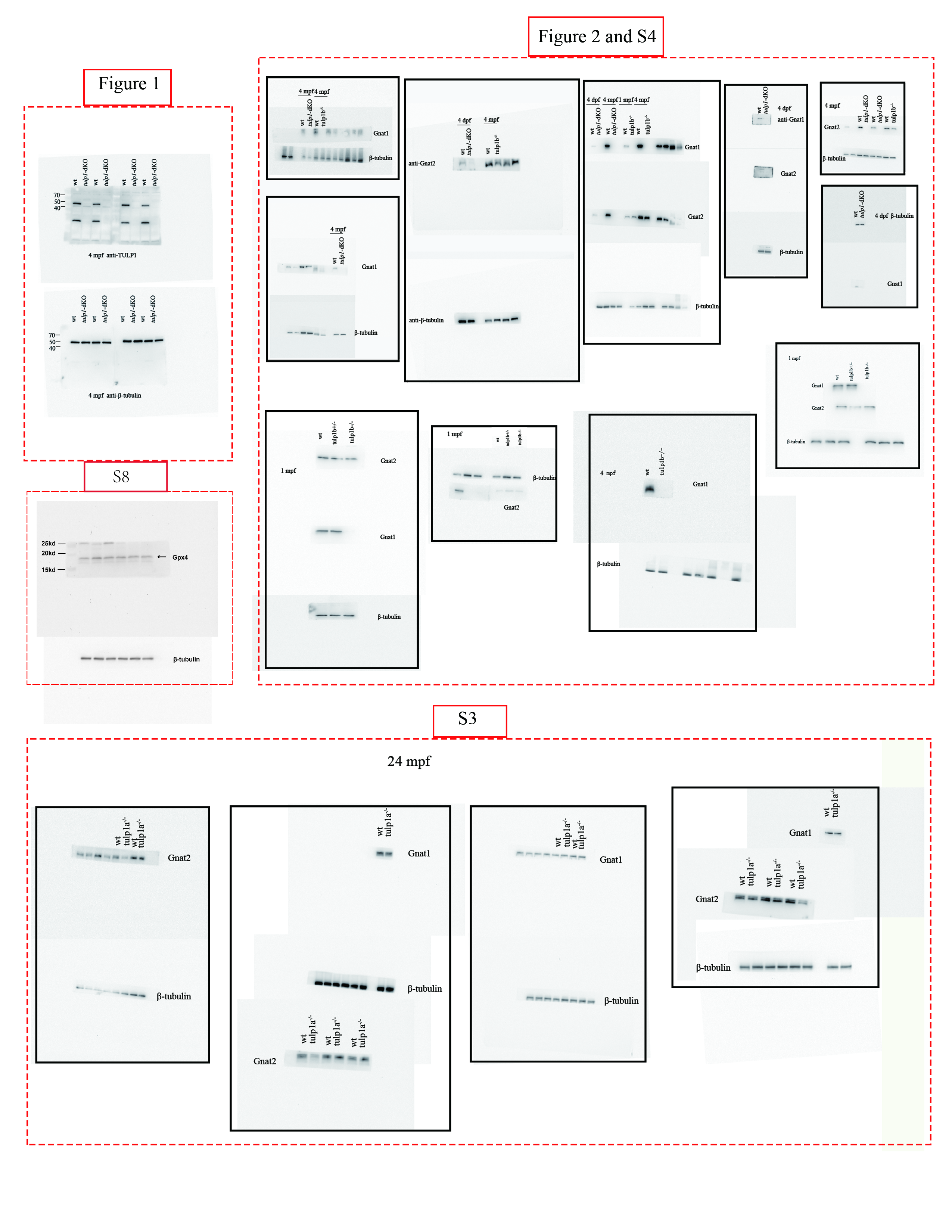

Supplement: Supplementary file 2 — Original western blots [file 41419_2022_5372_MOESM2_ESM.tif]
